# Correction: Borovikov et al. The Twisting and Untwisting of Actin and Tropomyosin Filaments Are Involved in the Molecular Mechanisms of Muscle Contraction, and Their Disruption Can Result in Muscle Disorders. *Int. J. Mol. Sci*. 2025, *26*, 6705

**DOI:** 10.3390/ijms27125491

**Published:** 2026-06-18

**Authors:** Yurii S. Borovikov, Maria V. Tishkova, Stanislava V. Avrova, Vladimir V. Sirenko, Olga E. Karpicheva

**Affiliations:** 1 Institute of Cytology, Russian Academy of Sciences, 4 Tikhoretsky Av., 194064 St. Petersburg, Russia; mariiatiskova@gmail.com (M.V.T.); avrova@rambler.ru (S.V.A.); sirw@mail.ru (V.V.S.); karpolka@bu.edu (O.E.K.); 2 Department of Pharmacology, Physiology and Biophysics, Chobanian and Avedisian School of Medicine, Boston University, 700 Albany St., Boston, MA 02118, USA

Missing Citation

In the original publication [1], ref. [62], “Borovikov, Y.S.; Tishkova, M.V.; Karpicheva, O.E. Twisting and untwisting of actin and tropomyosin filaments may be involved in the molecular mechanism of muscle contraction. *Biochem. Biophys. Res. Commun.* **2025**, *773*, 152080. https://doi.org/10.1016/j.bbrc.2025.152080”, was not included. Consequently, all subsequent references have been renumbered, and the in-text citation numbers have been updated accordingly. The citation has now been inserted into the following sections:

The Introduction (page 3, eighth paragraph) should read as follows:

In this study, polarization microfluorimetry was used to assess conformational changes in muscle fibers reconstructed with different isoforms and mutants of tropomyosin, troponin, and S1 at various stages of the ATPase cycle, with fluorescent labels on actin, tropomyosin, and myosin. Control experiments with wild-type tropomyosin isoforms were performed as previously described [62] and provided the baseline for interpreting the effects of point mutations. Changes in the twisting and bending stiffness of actin and tropomyosin filaments were detected, as well as the tilt of the myosin head relative to the axis of the actin filaments, which correlated with ATPase activity levels. We show that point mutations, such as Arg167His, Lys168Glu in Tpm1.1, Arg91Gly, Glu117Lys, and Gln147Pro in Tpm2.1 and Tpm2.2, Arg90Pro, Glu150Ala, and Glu173Ala in Tpm3.12, significantly alter the twisting of tropomyosin and actin, as well as their bending flexibility. This, in turn, leads to an increase in the calcium sensitivity of thin filaments and a decrease in actomyosin ATPase activity. These results suggest that the twisting and untwisting of tropomyosin and actin filaments, as well as changes in their bending stiffness, play a key role in the molecular mechanisms of muscle contraction.

The Results and Discussion (Section 2.1, page 4, third paragraph) should read as follows:

F-actin, tropomyosin, and S1 were labeled with fluorescent probes. Fluorescein isothiocyanate conjugated with phalloidin (FITC-phalloidin) was bound to F-actin in the actin groove (FITC-actin); 5-iodoacetamidofluorescein (5-IAF) was covalently linked to Cys residues of Tpm1.1, Tpm2.1, Tpm2.2, and Tpm3.12 (AF–Tpm); N-(iodoacetaminoethyl)-1-naphthyl-amine-5-sulfonic acid (1,5-IAEDANS) was specifically linked to Cys707 of S1 (AEDANS–S1). Incorporation of these proteins initiated polarized fluorescence, the measurement of which allowed for tracking changes in the spatial arrangement and flexibility of F-actin, tropomyosin filaments, and myosin heads during different stages of the ATPase cycle (Figure 1) [30,37,62,63,68,69].

The Figure 1 Legend (page 4–5) should read as follows:

**Figure 1.** The localization of the fluorescent probes 5-IAF (blue), FITC-phalloidin (green), and 1,5-IAEDANS (red) bound to tropomyosin (Tpm, blue), F-actin (pink), and myosin subfragment-1 (S1, yellow), respectively, in the reconstructed muscle fibers [62]. Gray arrows indicate the direction of polarized light emitted by the fluorescent probe, forming an angle Φ_E_ (emission angle) relative to the fiber axis (dashed line).

The Results and Discussion (Section 2.1, page 5, sixth paragraph) should read as follows:

It is known that the structure of tropomyosin is pre-shaped for binding with actin [80]. At the same time, during the contraction cycle, actin can change its conformation, rigidity, and twist angle [31,32,81]. Tropomyosin also undergoes conformational changes, moving along the actin surface. We hypothesize that changes in the bending stiffness of both proteins occur in a coordinated manner and are associated with the twisting of the filaments [62]. To assess the contribution of these changes to the ATPase cycle and force generation, we measured the orientation of fluorescent probes on tropomyosin and actin, as well as their bending stiffness in the presence and absence of troponin, calcium ions, and nucleotides. The incorporation of AF-Tpm of α-, β- or γ-isoforms or FITC-actin into ghost fibers, as well as the binding of AEDANS–S1 to F-actin initiated polarized fluorescence [30,37,48,64,68,77]. The polarized fluorescence intensity of four components was measured when the fiber was aligned parallel (_II_I_II_, _II_I⊥) and perpendicular (⊥I⊥, ⊥I_II_) to the excitation light’s polarization plane (see Materials and Methods). Employing the helical plus isotropic model [48] and the fluorescence polarization data allowed us to determine the angle between the emission dipoles and the fiber axis (Φ_E_), the bending rigidity (ε) for AF-Tpm and FITC-actin, as well as the relative number of disordered probes (N), along with Φ_E_ for AEDANS–S1.

The Figure 5 Legend (page 15–16) should read as follows:

**Figure 5.** (**a**–**d**) Sequential changes in filament twisting and untwisting at different stages of the ATPase cycle [62]. (**a**) Upon Ca^2+^ binding to troponin, actin filaments become twisted whereas tropomyosin filaments become untwisted, and their bending stiffness increases and decreases, respectively. (**b**) In the AM*•ATP stage, actin filaments become overtwisted and their bending stiffness increases, while tropomyosin strands undergo untwisting and their bending stiffness decreases. (**c**) During the transition to the AM•ADP stage, strong binding of the myosin head to actin is accompanied by actin untwisting and tropomyosin twisting. Correspondingly, actin stiffness decreases and tropomyosin stiffness increases. The extent of these changes correlates with the level of myosin ATPase activity. (**d**) In the relaxed state (in the presence of MgATP at low Ca^2+^), actin filaments exhibit greater twisting and their bending stiffness increases, while tropomyosin becomes untwisted and more flexible. The arrows indicate the direction of twisting or untwisting of the filaments. All structural transitions shown in the figure are schematic and not drawn to scale.

The authors state that the scientific conclusions are unaffected. This correction was approved by the Academic Editor. The original publication has also been updated.
